# Recomendaciones concretas para contrarrestar la información errónea durante la pandemia de COVID-19^*^

**DOI:** 10.26633/RPSP.2021.59

**Published:** 2021-05-13

**Authors:** Joan Donovan

**Affiliations:** 1 Escuela de Gobierno John F. Kennedy de la Universidad de Harvard Cambridge Estados Unidos de América Escuela de Gobierno John F. Kennedy de la Universidad de Harvard, Cambridge, Estados Unidos de América.

La pandemia de COVID-19 plantea retos multifacéticos para el sistema de salud estadounidense. Uno de esos retos consiste en dar información vital sobre salud al público, tarea que se ha hecho más difícil por el flagelo de la información errónea sobre la salud en todo el ecosistema de la información (1, 2). Aquí se formulan algunas recomendaciones concretas para los oficiales de la información de salud pública y los profesionales de la comunicación que preparan campañas de comunicación para los organismos y organizaciones de salud a fin de maximizar la posibilidad de que las advertencias de salud lleguen al público oportunamente.

En el Shorenstein Center de la Harvard Kennedy School, el proyecto de investigación de tecnología y cambio social estudia cómo se propaga la información errónea y cuál es su impacto en la política y la sociedad (bit.ly/2YcTX09). A diferencia de la desinformación política, o las noticias falsas, la información errónea sobre la salud puede dar lugar rápidamente a cambios en los comportamientos, razón por la cual los comunicadores de salud no pueden esperar a que las empresas de tecnología resuelvan el problema (3).

Por ejemplo, la investigación sobre los movimientos antivacunas revela cómo las celebridades, los activistas y los médicos desacreditados ganan influencia sobre las políticas de vacunación, al tiempo que también promueven la curandería, la información errónea y las conspiraciones en las redes sociales (4). Si bien es difícil saber quién se ha visto afectado por la información errónea sobre la salud, las mejores estrategias para contrarrestarla se centran en abordar a los “públicos silenciosos” con mensajes directos, cuidadosos y sucintos (5).

Los buscadores y las plataformas de redes sociales están luchando para controlar la ola de la nueva atención que se está prestando a la COVID-19 y están afrontando dificultades para hacer coincidir la información correcta con la persona correcta en el momento adecuado. Por ejemplo, una búsqueda en Google, Facebook, Twitter o YouTube del equivalente en inglés de la pregunta “¿dónde puedo hacerme la prueba del coronavirus?” tendrá como resultado información diferente, o peor aún, noticias falsas, una estafa depredadora o un *software* maligno (*malware*) (https://politi.co/3g9uzOE).

La pandemia deja al descubierto cómo el diseño algorítmico de los buscadores y las redes sociales, que priorizan los contenidos nuevos y pertinentes, contribuye a la confusión al mezclar diferentes tipos de información en un solo contenido inicial: lo mundano, lo relevante y recomendaciones médicas críticas (https://bit.ly/3iQoetq). Además, dado que muchas plataformas están diseñadas de modo que la publicidad es su columna vertebral, el contenido acreditado procedente de organismos de salud, profesionales de la salud y gobiernos locales a menudo se subsume en los anuncios publicitarios que buscan conseguir clics (6).

La situación es grave. Las personas necesitan información oportuna, pertinente y local sobre la COVID-19. Del mismo modo, los hospitales, los gobiernos, los organismos de salud y las universidades están abrumados por las consultas y necesitan utilizar la comunicación masiva para llegar a todos. Toda estrategia de comunicación debe recurrir a la redundancia de manera de difundir la misma información a través del mayor número de distintos canales que sea posible.

A continuación, se formulan cinco recomendaciones basadas en nuestra investigación realizada en el Shorenstein Center sobre la información médica errónea:

1. Los registradores de dominios han notificado más de 120 000 dominios con palabras clave relacionadas con el coronavirus o la COVID-19. Aunque la mayoría de los nuevos dominios no tienen ningún contenido, los estafadores están utilizando dominios personalizados para dirigirse a las personas que buscan información sobre tratamiento, las personas preocupadas que se encuentran bien y las que atraviesan dificultades financieras debido a la COVID-19 (https://nbcnews.to/3iT5QQu). Las organizaciones de salud pública y atención médica con sitios web ya establecidos y en funcionamiento no deberían registrar nuevos dominios porque es difícil ganar terreno dentro de los buscadores y las redes sociales. En su lugar, estas organizaciones deberían crear una página dedicada a la emergencia de salud específica, en este caso la COVID-19, en su sitio web ya existente y actualizarlo regularmente, incluso si no hay nada relevante que informar. Las actualizaciones proporcionan señales nuevas a los algoritmos, que las clasificarán en consecuencia.
2. Desmentir todos los rumores, todas las teorías de conspiración y todas las críticas políticas agota los recursos esenciales. Además, ha habido un diluvio de solicitudes de entrevistas a personal médico y defensores de la salud pública. Los comunicadores de salud deberían establecer un protocolo de vigilancia para decidir qué información errónea está ganando terreno y acercándose a un punto de inflexión, como cuando dicha información se propaga a través de plataformas o la disemina una persona que despierta interés periodístico, como un político o una celebridad. Recomendamos consultar regularmente la base de datos de rumores de la Agencia Federal para el Manejo de Emergencias (https://bit.ly/3k-SOKUO) y la base de datos de comprobación de los hechos de Google que contiene noticias recientemente desmentidas (https://bit.ly/2Ebnwbg); examinar los comentarios publicados en grupos de redes sociales locales y aplicaciones de mensajes públicos, como Nextdoor; y mantener un registro de los comentarios que recibe la organización a través de sus cuentas de redes sociales, las líneas telefónicas o el correo electrónico. Es importante destacar que nadie debe responder a la información errónea a menos que haya buenas razones para hacerlo y se tenga un plan para comunicar la respuesta públicamente (https://bit.ly/3j4PKnh). Se recomienda no responder a las personas, sino más bien desmentir los principales temas de información errónea.
3. Satisfacer la demanda de nueva información durante esta pandemia requerirá un cambio hacia estrategias de comunicación masiva. En términos de comunicación de riesgos, trabajar con periodistas es la clave para luchar contra la información errónea. Tender puentes de comunicación bidireccional entre los comunicadores de salud y los periodistas locales asegurará la visibilidad y la confianza en todos los sectores profesionales cuando ocurran emergencias de comunicación. Esto no es lo mismo que organizar ruedas de prensa. Se trata de forjar relaciones verdaderas, en las que la salud pública sea el objetivo común. Ayudar a los periodistas a desmentir la información errónea y dar recomendaciones clave aumentará la credibilidad y la visibilidad de las recomendaciones de salud pública dirigidas a amplios públicos.
4. Si utilizan las redes sociales para comunicarse, algo que deberían hacer todas las organizaciones de salud pública, es importante ponerse en contacto con las plataformas y solicitar publicidad gratuita de servicio público. En una crisis como esta, los sistemas de publicidad en línea se pueden readaptar para llegar a los públicos locales (https://bit.ly/3gcHpfc). Las noticias de la televisión local siguen siendo una forma fiable de informar a muchas personas de manera rápida y local.
5. Los gobiernos locales y los organismos de salud deben establecer sistemas de mensajes de texto y notificaciones de SMS (servicio de mensajes cortos, por su sigla en inglés), siempre que sea posible, para llegar a las personas fuera de las redes sociales. Aunque en la gestión de emergencias se recomienda encarecidamente que los gobiernos establezcan estos sistemas antes de un desastre, la pandemia es una oportunidad para inscribir a muchas personas. Alternativamente, los sistemas de alerta de emergencias no requieren ningún registro y podrían adaptarse para llegar a las personas en una zona geográfica determinada. Por ejemplo, la ciudad de Nueva York ha utilizado alertas de emergencias para solicitar los servicios de trabajadores de atención de salud.

En este momento, las empresas de búsqueda y redes sociales no están diseñadas para proporcionar información autorizada, oportuna, pertinente y local. Las empresas de tecnología se encuentran en una encrucijada, en la que las alianzas y coaliciones establecidas ahora para hacer frente a la pandemia de COVID-19 configurarán el futuro de la comunicación de riesgos en la internet. Por lo tanto, es esencial que los profesionales de la comunicación de salud comprendan las limitaciones de las redes sociales y trabajen activamente para mitigar la información errónea y aminorar los daños causados por estafas, conspiraciones y engaños; el público debe poder tener acceso a información oportuna, local y pertinente cuando más la necesite.

## Declaración.

Las opiniones expresadas en este manuscrito son responsabilidad del autor y no reflejan necesariamente los criterios ni la política de la *RPSP/PAJPH* y/o de la OPS.
